# Challenges for food security and safety: a qualitative study in an agriculture supply chain company in Iran

**DOI:** 10.1186/s40066-021-00304-x

**Published:** 2021-10-07

**Authors:** Ahmad Kalateh Sadati, Mehdi Nayedar, Leila Zartash, Zahra Falakodin

**Affiliations:** 1 Department of Social Sciences, Yazd University, University Blvd, Safayieh, PO Box: 98195-741, Yazd, Iran; 2 Department of Animal Science, Tehran University, Tehran, Iran

**Keywords:** Food security, Food safety, Agricultural development, Company survival

## Abstract

**Background:**

Food supply chain companies are major link for safe food production and distribution. Food safety ensures reduced food losses and, therefore, contributes to food security. Although there have been extensive studies on the food industry with different perspectives, no study has so far been conducted on the challenges in terms of the food security. The present study is a qualitative one conducted in 2019 in one of the largest agricultural production chain companies in Khorasan Razavi province, Iran.

**Results:**

The research method was based on the conventional content analysis. Triangulation method was used for the data collection being a combination of the participant observations, focus group discussions (17 managers) and semi-structured interviews with the president of the company. Participants believed that their company plays an important role and has a prestigious position in the food security of Iran. However, the main challenges of the company are *regulatory system, food safety risks, market unpredictability, traditional management* and *sanctions*.

**Conclusions:**

The company is highly resilient to all these challenges; however, the traditional management is still a serious disadvantage. This leads to the managers’ burnout along with the company survival problem, removing unfair sanctions and protecting agricultural development in developing countries is a necessity. In general, paying attention to the modern organizational management of these companies and their survival is an essential policy. For this reason, it is suggested that trust must be established between policymakers and companies on one hand and relationship between the academia and such companies to promote the organizational management on the other hand.

## Background

An adequate food security is the main issue [33–35] because there is a global demand for food [14]. The World Food Summit [38] declares that: “Food security exists when all people, at all times, have physical and economic access to the sufficient, safe and nutritious food which meets their dietary needs and food preferences for an active and healthy life” [38]. Four main elements of food security are the availability, stability, utilization and access [4, 36].

Whether we can feed the world is a question arising in the food security [16, 40] especially in developing countries. In these countries, food insecurity is so vital since it is related to the intake of a number of unhealthy dietary items among the poorest households in urban India [30]. In addition, it leads to the global well-being inequality, with the youth in poorer countries having poor well-being in terms of food access and poverty [23]. Lower income regions face with the population growth, lack of adequate food resources, traditional farming, low technology, lack of knowledge, and shortage of trained and skilled human resources. Therefore, various mechanisms are associated with food availability, food access and environmental impact [20]. For example, one of the new agricultural threats to Africa is that farmers are turning into non-agricultural activities such as illegal small-scale mining [2]. It seems that the commercial and trade interest in a few countries hamper the welfare of millions of poor people living in developing ones [37]. These conditions lead to extreme poverty, malnutrition and hunger which are major issues in many developing countries [12]. Therefore, food security is linked to the consumption, production, and marketing of food, the functioning of factor markets especially for labor social safety nets, governmental and nongovernmental assistance agencies, initial asset and income distributions, and myriad other subjects across several disciplines [6].

Food security faces many challenges, from the introduction of fertilizers containing phosphorus, nitrogen and potassium to the agricultural fields [8] to falling water tables and rising temperatures [16, 22] and continuing population and consumption growth [14]. However, the global inequality and capitalist forms of production and distribution appear to be the major challenges to food security. Therefore, we are all encountered with a capitalist food system and need to know about capitalism [18].

Food safety and security in Iran is also related to several issues such as society, culture and consumption. Iranian society has faced issues such as sudden population growth, severe climate change and prominent, social and cultural changes in recent decades. Urbanization, lifestyle and social and cultural changes are the challenges to the food security in Iran. In a study conducted by Sadati, it was found that lifestyle changes have become a major problem, leading to the food waste in Iran [32].

In addition to the nutritional and phytochemical potential of sorghum in food processing for food security [10], the importance of health in related food chains is not hidden from anyone. For example, Panghal et al. has pointed out the importance of current malpractices followed by retailers to mislead the consumers about fruits and vegetable quality using sweeteners, colors and other chemicals in post-harvest malpractices for fresh fruits and vegetables [29]. To promote the food safety India has introduced the food safety and Standards Act 2006 to overcome the deficiencies in the Prevention of food adulteration act, 1954 [28]. In addition, it was proposed that traceability continues as a main tool for controlling the food safety in food chain [11].

Food safety and food security are interrelated concepts. Food safety must be an enabler and not inhibitor of global food security [21]. Coordinated efforts are needed to increase the production of food but with a view to enhanced food quality and safety as well as to controlling residues of persistent pesticides in the environment [7].

Food production is an important aspect of food security [27] and food safety [10]. Despite many problems, the agricultural and especially alternant industries has been growing relatively well in recent decades. Food industries especially poultry are pivotal in Iran as well as in other societies. That is why policy makers have emphasized the production infrastructures in different provinces of Iran and focused on enhancing the sustainable production [3].

The poultry industry of the country needs further investigations, especially the challenges that these industries face. There is still a lack of knowledge on this issue. In addition, despite effect on these chain after coronavirus, it seems that they are the main items of food security in the time of COVID 19. Due to the importance and status of these industries, the present study aims to evaluate the meaning and concept of food security from the perspective of managers of one of the major poultry industries in Iran. The main purpose of the research is to analyze the food security challenges from the perspective of the company managers.

## Materials and methods

The present study is a qualitative one conducted in 2019 in, Khorasan Razavi province, Iran. The study was conducted in a private food chain company as the largest one in Khorasan Razavi province. It has about 600 workers and operates in broad areas, including the food-animal production, poultry slaughter and associated cold store, breeding poultry farm, one-day old broiler chicks, egg production farm (1 ton daily) and milk collection system. In addition, the company has a very wide distribution system nationwide, distributing its products in all of the above-mentioned fields.

The triangulation method was employed for the data collection. At first, data were gathered with three focus group discussions. One focus group which took place at the company’s livestock agriculture and animal feeding, was attended by three mid-level, three sales and one senior managers. The second meeting was held at the company’s chicken slaughter section. Here, the focus group included five individuals, including a senior advisor to the company president, a senior manager of the slaughterhouse and three other senior staff. The final focus group was centralized on the production center of the mother poultry and egg farms, attended by five managers, and in particular the senior quality control manager of the company. In addition, an in depth-interview was conducted with the president of the company. According to Hennink et al. [17] we reached to saturation based on 3 items which are study purpose (which reached it), type of codes (in line of study porous), and group stratification with verity of company managers.

The main question in these discussions and interview were ‘‘As one of the largest food production industry in the country, what challenges do you face?’’, ‘‘What are the threats to the food security?’’ and, ‘‘How have you been able to cope with these challenges?’’ Based on the participants’ responses, the questions such as “could you explain more” were applied to broaden the scope of the discussion. In the focus groups, we also asked for other participants’ opinions to find whether they agreed about the ideas of other participants.

Furthermore, field observations were conducted to achieve rich data. Accordingly, with the assistance of a Ph.D. student in sociology, field observations in the company area were conducted during 4 days. The purpose of the field observations was to evaluate the interactions and practices of the company staffs and workers on a regular and daily basis. These observations provide a great deal of support for researchers in analyzing the interview data.

The data transcription was carried out using qualitative content analysis. This method analyzes the social phenomena in a non-invasive nature and focuses on interpreting and describing the topics and themes meaningfully. Based on Hsieh and Shannon, there are three main approaches of content analysis: conventional, directed and summative. In the conventional approach, the coding categories are directly derived from the text data [19]. For this reason, four steps were taken in the current study which include defining the unit or theme of analysis, developing categories and coding scheme, coding all the text and drawing inferences on the basis of coding or themes. This process was performed in a reciprocal path between the coding and data.

Code consistency was verified by the research members. Also, member checking and transferability of the code were conducted to assess the validity and reliability. The ethics of Helsinki Declaration and American Sociological Association were the focus of the researchers’ attention.

## Results and discussion

All participants believed that the company plays a pivotal role in the food security of Iran. The high production volume, use of advanced technology, high quality products and high resilience of the company proves this claim. The company has been providing significant support, especially during the sanctions time and when meat is scarce over the country. In these situations, it has come to the aid of the community by supplying egg and meat. In addition, all participants emphasized the *QUALITY* of their products which is related to food safety. The products including animal feed egg and chicken meat are produced with high quality in this company. They control the raw materials, production processes and finally their food supply trend in the market. However, there are challenges that threat their company and food security as well. Since this company is a chain, therefore it is more resistant to the challenges and threats and controls the environmental pressures with a high resilience. However, the company is under many pressures such as *regulatory system, consumption culture, market unpredictability, sanctions* and *traditional management*. Participants believed that the food industries in Iran face such challenges. Some petty farming industries are going bankrupt due to these obstacles and challenges. Hence, food security requires more attention to these challenges.

### Regulatory system

Participants emphasized high quality of the company’s products as a main strategy. When production is to meet high quality, its costs go up. Therefore, the company has to increase the final price. However, the surveillance system sets a price ceiling which can cause company losses.

We give the best foods to our chickens. Therefore, the quality of our eggs and meat is high. One of our concerns in the market is the price issue (one of the high-level managers).

However, the animal food production does not face this problem since consumers know that high quality food yields better productivity. In these cases, the control system also monitors, and customers are willing to purchase high quality animal feed, though they are more expensive. However, the company cannot set prices based on the quality. The egg and meat production with high quality and therefore higher prices, is the main concern of the surveillance institutions. They require the company to deliver its’ products at a set price and that is a huge loss for the company. In some cases, the quality challenge faces more serious obstacles and conflicts of interest in the marketplace. In these cases, the stakeholders do not allow the company to introduce its brand as high quality products. For example, the company insists that the eggs be ‘antibiotics free’, but stakeholders have pushed through relevant organizations to remove the labels from the product packages.

Our products are ‘antibiotic free’. We spent about $ 12,000 on the labels to introduce our products being free of antibiotics. However, they came from relevant organizations and forced us to cut the labels because of conflicts of interest. All our money was wasted (selling manager).

One of the most important challenges to the regulatory system is exports. Exports are prohibited where meat and egg supply in the country is hampered. Therefore, they lose their foreign customers in the competitive market of the Middle East.

We had good egg customers in Afghanistan. In the time of price control, export was banned, we lost our customers and they replaced Pakistani exporters (high-level sales manager).

### Food safety risks

Under the poor economic conditions, price determines the consumption behavior. Therefore, consumption culture is determined by the socio-economic variables which manage their food safety. This is more important in the competitive situations where price matters rather not the quality. While competitors produce low-quality meat and eggs with lower costs, consumers tend to buy cheaper products. In a competitive market, the company has to make great efforts to maintain the quality of its products.

On our chicken farms, we provide the best food with the best supplements. Consumers do not care about this as they are looking for the cheaper food. Chickens raised in most places do not know what to eat. Sometimes they are given junk. This affects their meat and eggs. But people and their culture do not pay attention to this issue due to culture and economic problem. It does not matter which farm the eggs are produced in or the type of food they have bred with, whereas these factors may affect the quality of meat and eggs (president of the company).

Another cultural issue is that people prefer obese chicken than the medium one in Iran. However, obese chickens have a lot of fat and this is very harmful to human’s health. This fat is mostly discarded in the kitchens. The tendency to buy obese chicken and waste it is a big economic issue in Iran. The breeding process takes about 10 to 15 days longer to produce obese chickens according to the family expectations. Here, billions of dollars are wasted in production and social system.

Our people think that fat is better. They prefer to buy chicken more than 3.5 kg. However, these chickens have a lot of fat which is harmful to the health and must be discarded. Chicken should be delivered within 34 days weighing about 2.5 kg. Billions of dollars are then spent on breeding fat chickens and wasting fats is a futile job (boss of the company).

### Market unpredictability

Production in the country needs stable conditions. Food producer must be aware of the market conditions and be able to predict it. Market unpredictability is an important point which sometimes leads to the producer bankruptcy.

The producer does not know whether his production is doing well in the market or not. It's not just about the products, we are confused even when we decide to import technology. (a mid-level manager).

The main cause of unpredictability is the currency rate changes. This problem significantly affects buying the raw food material and food technology.

Sometimes we want to order raw materials or devices for the company. However, we are not sure whether to order with this currency market or not. Nothing is definite (company accountant).

Much of this unpredictability is related to the international issues, unfair pressures and sanctions. As stated in the regulatory pressure theme, unpredictability about the costumers, especially, those abroad is the main problem the company faces with. While marketing managers try to attract customers, market instability and especially social issues, drives the customers out of the sales list.

### Sanctions

An important issue which threatens the food security is the international sanctions. Unfair sanctions are the main pressures on the company, specifically at this moment that Iran is subjected to international sanctions imposed by a number of countries. The company imports the best type of corn to obtain high quality animal feed. However, importing high quality corn have been severely affected by the sanctions.

We have problem with importing high quality corn. We do not really know what to do at this moment. On one hand, we do not want to lose our customers and on the other hand, we have to emphasize the QUALITY. In this situation, we made attempts to cope with this problem (one of the managers).

Moreover, the company uses dollar currency for its imports. The sanctions have caused sharp fluctuations in the currency price and the company cannot make a clear decision on importing raw material for the animal feed industry or technology.

We wanted to import a high quality corn consignment from Brazil. When we encountered with the sanctions, we had to deal with a lot of problems and had to use our stored resources. Resources that were running out (sales manager).

The other problem the company faces with in this condition, is the technology importing. The company not only cannot import the high-tech industrial products or materials, but it also has problems in importing low technology. In addition, developed countries never provide developing ones with up-to-date knowledge.

We sometimes have to import used devices due to the sanctions. Developed countries never share their knowledge with us. It is quite clear that this is their strategy. Even in the face of sanctions, their low-level knowledge is withheld from us (high-level manager).

### Traditional management

Traditional management is another problem with which the company is encountered with. A company with about 600 workers has been run in the same traditional way. This has, in many cases, raised problems or controversies within the company to be resolved by the boss. This led to the diminished authority of the head of the staff, especially the new ones. The company lacked modern organizational system and therefore the relationships and interactions were generally driven by traditional approaches in a face-to-face manner.

One of our problems is our staff. I spend a lot of time with our weak staff, I trained and recommended them to work well. It wastes some of our energy and takes time (boss of the company).

Traditional management is prevalent everywhere. This has led to the verbal transference of needed skills from a worker to another, not through the organizational management training or elective systems.

We need education. Currently, we only receive oral training from the predecessors. The company needs to formalize and train its employees (manager of the quality section).

The survival of a company depends on the extent to which it uses modern organizational management. This can also affect the future of the company. Researchers asked the boss of the company about the company’s future. He did not make a clear response and said ‘*I’m trying to prepare my older son for the future situations*’. The president of the company was not familiar enough with the modern organizational system. However, he was optimistic that the company will survive under the family conditions and common interests.

## Discussion

Global food security will remain a worldwide concern for the next 50 years and beyond [31]. Food security is a major topic in academic and international debates [34] Agricultural production companies are the basis of this security. A great interest is devoted to the changes in consumers’ preferences and expectations as well as to the analysis of food innovations and their impact on the global market [35]. The current study illustrated that food security in Iran is affected by national and international issues. At the national level, issues such as the regulatory system, consumption culture, market unpredictability and traditional management are the major obstacles. At the international level, unfair sanctions are a big challenge even affecting the internal affairs of the company.

The managers of this study were not looking for unfair prices that pressured the public, but expected the government to take into account the public interest in the price controls as well as the interests of agricultural production companies. Although the investigated company can survive the price volatility, petty agricultural companies may not have such capability. This finding is consistent with the study indicating that the government laws are closely linked to the food security, from energy and water consumption [9] to genetic resources [25] and food distribution.

*Food safety risks* was the cultural issue that the company needs to produce and sell fat chickens. The president of the company thinks that much of the money and material resources would be saved if this cultural taste is changed. This cultural issue requires serious social activities. As is the case with food waste [32], it seems that discussing Iranian lifestyle requires serious cultural work. Olum et al. indicated that the cultural restrictions applied particularly to the women and children over the consumption of several nutritious foods, lead to the food insecurity in Uganda [26]. The multi-level dimensions of power must be viewed as they are related to the individuals, households and broader community dynamics that are essential to the understanding of the local dynamics of food security [39]. Therefore, the food security policy can be improved through taking the culture into account more than ever [4].

Other *food safety risks* item was the consumer’s consumption which does not care about the quality. The fact is that this item follows most of the socio-economic components. As reported before, income alongside other variables significantly explain the consumption behaviors [2]. However, research has indicated no effect on the organic food consumption behavior [13].

About the *food safety risks* the head of the factory pointed out that part of the production of chicken meat and eggs is not done by observing the hygienic points and also the appropriate food for the chickens. Nevertheless, such cheap products have a better market. One part is related to people’s culture and the other part is related to economic issues. He suggested that more monitoring be done to promote food safety. This is in line with suggestions of other scholars about promotion of food safety [10, 28]. This finding is in line with traceability continues as a main tool for controlling the food safety in food chain [11].

Another important internal threat was the traditional management and survival issues. The company has not yet made any serious decision on these issues. It means that it is vulnerable to the internal changes despite its high resiliency. This is in line with Grashuis’s recommendation about the survival of farmer cooperative in USA [15]. The role and high-level position of the present agricultural production company has also linked its future with the food security issue in Iran, which requires more attention from policymakers.

Finally, one of the most significant threats to the food security in the country is the unfair international sanctions. These sanctions impose a lot of pressure on the country’s food production industry, even though those industries and companies are highly resilient. Participants believed that bankruptcy of small companies is due to the impact of existing sanctions. In addition, sanctions prohibit buying hi-tech agricultural industries for Iran. Undoubtedly, supporting Iran’s food industries is support for manufacturing industries worldwide. Food security is a global chain, pushing one part of which, affects the other loops. This is part of the item that many scholars called it right to food [5, 24, 41]. A multifaceted and linked global strategy is needed to ensure sustainable and equitable food security, different components of which are explored here [14]. Right to food [41] is the most important concern for developing countries, including Iran. In developing countries, the technological innovation plays an important role in the sustainable agricultural development [1].

## Limitations and suggestions

This is a qualitative study with non-generalization feature. Conducting quantitative study in this path is proposed. Future studies on the survival of agricultural production companies are suggested in terms of the research and policy making.

## Conclusion

Food security and food safety in developing countries are a complex phenomenon. Despite the internal resistance of the case study, it is clear that our food production is are vulnerable to the internal and international conditions. Regulatory system, culture, unpredictability of the market, traditional management and sanctions are the main threats. The present case study was highly resilient, however it seems that other petty companies do not have such resiliency. However, paying attention to the technological agricultural development along with removing the sanctions is of high necessity. Finally, food safety, needs using regulatory system along with a promotion in culture and solving financial issue.

## Data Availability

Due to the confidentiality of interviews and authors commitments to interviews, we cannot make available transcripts, but the table of analysis and interpretation the data would be available on demand.
